# Implementing a perioperative weight management program for ovarian cancer patients undergoing ultra-radical cytoreduction: a quality improvement initiative

**DOI:** 10.3389/fonc.2026.1865561

**Published:** 2026-07-22

**Authors:** Chenlu Ying, Wei Cao, Tingting Fan, Liying Fu

**Affiliations:** Zhejiang Cancer Hospital, Hangzhou Institute of Medicine (HIM), Chinese Academy of Sciences, Hangzhou, Zhejiang, China

**Keywords:** chemotherapy timing, cytoreductive surgery, multidisciplinary care, nutrition, ovarian cancer, skeletal muscle

## Abstract

**Background:**

Patients undergoing ultra-radical cytoreductive surgery (CRS) for advanced ovarian cancer are at high risk of postoperative catabolic stress, leading to unintentional weight loss and skeletal muscle depletion. Structured perioperative nutrition and rehabilitation pathways remain insufficiently described in this setting.

**Objective:**

To evaluate the association between a multidisciplinary perioperative weight management program and pre-chemotherapy postoperative weight loss, nutritional status, body composition, and treatment-related recovery among patients undergoing ultra-radical CRS for ovarian cancer.

**Methods:**

This quasi-experimental quality improvement study included 160 patients undergoing ultra-radical CRS at a tertiary oncology center. Patients treated between January and December 2024 received routine care (control group, n=80), whereas patients treated between April and October 2025 received a structured multidisciplinary intervention (experimental group, n=80). Allocation was based on treatment period rather than randomization. The primary outcome was pre-chemotherapy postoperative weight loss rate. Secondary outcomes included changes in serum albumin levels, body composition parameters, time to initiation of adjuvant chemotherapy, and postoperative length of stay.

**Results:**

The experimental group showed a significantly lower weight loss rate compared with the control group (2.90% vs. 5.31%, P<0.001). The proportion of patients with clinically significant weight loss (>5%) was also significantly lower in the experimental group (31.3% vs. 48.8%, P = 0.024). The decline in serum albumin was significantly attenuated (−4.10 vs. −6.98 g/L, P<0.001). Fat-free mass showed an apparent increase in the experimental group and should be interpreted cautiously because bioelectrical impedance-derived lean-mass estimates may be influenced by perioperative fluid shifts. Skeletal muscle loss was numerically smaller but did not reach two-tailed statistical significance after correction (P = 0.055). Time to initiation of adjuvant chemotherapy was shorter in the experimental group (14.84 vs. 22.03 days, P = 0.002).

**Conclusion:**

A multidisciplinary perioperative weight management program was associated with lower pre-chemotherapy postoperative weight loss, better preservation of selected nutritional indicators, and earlier readiness for adjuvant chemotherapy. Because of the historical-control design, restricted BMI range, and incomplete oncological outcome data, these findings should be interpreted as hypothesis-generating and require confirmation in prospective multicenter studies.

## Introduction

Ovarian cancer (OC) remains the most lethal gynecological malignancy worldwide. According to the Global Cancer Statistics (GLOBOCAN) 2022, an estimated 324,603 new ovarian cancer cases and 206,956 deaths occurred worldwide; Asia accounted for 178,223 new cases (54.9%) and 109,547 deaths (52.9%) (1). In China, 61,060 new ovarian cancer cases and 32,646 deaths were estimated in 2022, making the disease a major regional public health challenge (2). Due to its insidious onset and the absence of effective early screening biomarkers, the majority of patients (approximately 70%) are diagnosed at an advanced stage (FIGO III–IV) (3). At this stage, the primary therapeutic objective is to achieve optimal cytoreduction during cytoreductive surgery (CRS). Attaining R0 resection remains one of the most important surgical prognostic factors for progression-free and overall survival (4).

To achieve this objective in advanced cases, surgical practice has increasingly shifted toward ultra-radical procedures (5, 6). These procedures extend beyond standard pelvic surgery and may involve upper abdominal and multivisceral resections, including bowel resection with anastomosis, splenectomy, distal pancreatectomy, diaphragmatic stripping or resection, extensive peritonectomy, and selected hepatobiliary procedures (5, 6). Although maximal effort cytoreduction can improve oncological outcomes, it imposes substantial physiological stress. Surgical complexity is clinically important because procedures such as diaphragmatic surgery, splenectomy, and bowel resection have different inflammatory and nutritional consequences (7).

Extensive tissue trauma can trigger a systemic inflammatory response and a hypermetabolic state characterized by accelerated proteolysis and lipolysis (8). This postoperative catabolic response is further exacerbated by gastrointestinal dysfunction, including prolonged ileus, early satiety, nausea, and impaired nutrient absorption, leading to a negative energy and protein balance (9–11). As a result, patients undergoing ultra-radical surgery are at high risk of unintentional weight loss (UWL), skeletal muscle depletion, and functional decline (12, 13). UWL and malnutrition have been associated with increased postoperative complications, reduced tolerance to adjuvant platinum-based chemotherapy, delayed recovery, and impaired quality of life (9, 11, 14).

Despite the recognized importance of perioperative nutritional management, current clinical protocols often lack a structured, evidence-based approach tailored to the specific metabolic demands of ultra-radical ovarian cancer surgery (9, 10, 15). A baseline clinical audit conducted at our institution (January-December 2024) revealed that 77.5% of patients experienced a weight loss greater than 2.4% before initiation of adjuvant chemotherapy. This local audit, together with existing evidence on perioperative malnutrition in gynecologic oncology, highlighted a gap between routine perioperative care and the nutritional needs of this high-risk population (12, 14, 15). Therefore, this quality improvement initiative was developed to evaluate a structured multidisciplinary perioperative weight management strategy for patients undergoing ultra-radical CRS.

## Methods

### Study design and rationale

This study was designed as a quasi-experimental quality improvement initiative using a historical control comparison. The intervention was implemented under a Plan–Do–Check–Act (PDCA) framework to standardize perioperative weight management (16). A randomized controlled trial was not used because the intervention was introduced as a hospital-wide quality improvement protocol after the baseline audit, and withholding a feasible nutrition-rehabilitation pathway from eligible patients during implementation was considered impractical in routine clinical care. Therefore, allocation was determined by treatment period rather than randomization.

Patients treated between January and December 2024 received routine perioperative care (control group), whereas patients treated between April and October 2025 received the multidisciplinary intervention (experimental group). Recruitment, eligibility screening, surgical indication assessment, and perioperative data collection procedures were conducted using the same institutional workflow in both periods. To reduce temporal confounding, patients were managed in the same tertiary oncology center by the same core gynecologic oncology service, and no planned changes were made to surgical indications, anesthesia protocols, postoperative analgesia, enhanced recovery routines, or criteria for starting adjuvant chemotherapy other than the weight management program.

### Participants

Eligible participants were adult patients (≥18 years) with pathologically confirmed ovarian cancer undergoing primary ultra-radical CRS. Inclusion criteria required the ability to provide informed consent and a baseline body mass index (BMI) between 18.5 and 24 kg/m². This BMI restriction was used to reduce heterogeneity in bioelectrical impedance analysis and to focus the initial quality improvement cycle on normal-weight patients, in whom early catabolic weight loss may be less clinically apparent than in underweight patients. However, this criterion also limits external validity to overweight or obese patients and is acknowledged as an important limitation.

Exclusion criteria included cognitive impairment, psychiatric disorders affecting treatment compliance, severe metabolic comorbidities (e.g., uncontrolled diabetes, advanced hepatic or renal dysfunction), and an estimated life expectancy of less than three months.

### Operational definition of ultra-radical CRS and surgical complexity

Ultra-radical CRS was operationally defined as primary cytoreductive surgery requiring at least one procedure beyond standard hysterectomy, bilateral salpingo-oophorectomy, omentectomy, and pelvic/para-aortic lymph-node assessment (5). Qualifying procedures included bowel resection or stoma creation, diaphragmatic stripping or resection, splenectomy, distal pancreatectomy, upper abdominal peritonectomy, partial hepatectomy or hepatobiliary procedures, and other multivisceral resections performed to achieve complete macroscopic cytoreduction. Surgical complexity was recorded as low, medium, or high according to the institutional surgical grading system, which was aligned conceptually with the Surgical Complexity Score categories used in ovarian cancer surgery (low, intermediate, and high complexity) (7).

### Multidisciplinary team collaboration

A multidisciplinary team (MDT) was established to implement the weight management program. The team included gynecological oncologists, clinical dietitians, specialist nurses, and rehabilitation therapists, coordinated by a senior nurse manager. Oncologists were responsible for surgical and medical management, dietitians conducted nutritional assessments and developed individualized nutritional plans; specialist nurses coordinated daily implementation and monitoring, and rehabilitation therapists designed and supervised functional recovery programs. Regular team meetings and training sessions were conducted to ensure protocol adherence.

### Perioperative weight management pathway

The intervention was delivered through a structured, risk-stratified clinical pathway comprising preoperative assessment, early postoperative reassessment, nutritional intervention, rehabilitation, and dynamic monitoring. The description was expanded according to the Template for Intervention Description and Replication (TIDieR) principle that interventions should be reported with sufficient detail to allow replication (17).

### Preoperative assessment

Upon admission, patients underwent comprehensive baseline evaluation, including body weight, body composition, handgrip strength, frailty assessment, and gastrointestinal symptom screening. Nutritional risk was assessed using the Nutritional Risk Screening 2002 (NRS-2002) (18). Body composition was measured by bioelectrical impedance analysis under standardized ward conditions whenever clinically feasible.

Patients were classified as high-risk if they met any of the following criteria: NRS-2002 score >3, clinically significant ascites, hypoalbuminemia, frailty score ≥1, or severe gastrointestinal dysfunction. High-risk patients were referred to the dietitian before surgery for individualized nutrition planning.

### Nutritional protocol

The nutritional protocol aimed to provide 25–30 kcal/kg/day of energy and 1.2-1.5 g/kg/day of protein, adjusted according to renal/hepatic function, gastrointestinal tolerance, inflammation, and surgical recovery (11). Oral feeding was preferred when tolerated. Oral nutritional supplements were recommended when dietary intake was estimated to be <75% of target intake. Enteral nutrition was considered when oral intake remained <60% of target for more than 48–72 hours and the gastrointestinal tract was functional (10). Supplemental or total parenteral nutrition was considered when enteral/oral feeding was contraindicated or insufficient because of ileus, severe intolerance, or postoperative complications. Dietitians reviewed high-risk patients within 24–48 hours after surgery and reassessed nutritional plans at least twice weekly or whenever weight loss thresholds were triggered.

### Postoperative risk stratification

Postoperative care was guided by a dynamic, risk-stratified framework based on weight loss relative to admission baseline. The three-level classification (<2%, 2-5%, and >5% weight loss) was developed by the MDT for this local quality improvement protocol, informed by clinical thresholds commonly used to identify early nutritional deterioration and clinically significant weight loss in oncology care (19). The categories were used as action thresholds rather than diagnostic criteria for malnutrition.

Patients in the low-risk group (weight loss <2%) received routine nutritional education, encouragement of early oral intake, and monitoring of body weight, dietary intake, gastrointestinal symptoms, and fluid balance every three days.

Patients in the moderate-risk group (weight loss 2–5%) received targeted nutritional counseling and systematic evaluation of inadequate intake, nausea, vomiting, ileus, diarrhea, pain, early satiety, or metabolic abnormalities. Dietary modification included small frequent meals (5–6 meals/day), high-protein food prioritization, oral nutritional supplementation when necessary, and symptom-directed pharmacological management. Patients in the high-risk group (weight loss >5%) triggered formal MDT review within 24 hours. Dietitians, nurses, physicians, and rehabilitation therapists jointly adjusted the nutrition and recovery plan using body composition, gastrointestinal tolerance, laboratory tests, and clinical status.

### Rehabilitation protocol

Rehabilitation began as early as clinically feasible. On postoperative day 1, patients were encouraged to perform breathing exercises, ankle-pump exercises, bed mobility, and sitting out of bed with assistance. Progressive ambulation was introduced after hemodynamic stability was achieved. From postoperative days 2-5, patients were encouraged to walk 2–4 times daily as tolerated and to perform low-intensity lower-limb strengthening and range-of-motion exercises under therapist or nurse supervision. Intensity was individualized according to pain, fatigue, drainage tubes, hemoglobin level, and surgical restrictions, with a target of light to moderate exertion. Exercise was withheld or reduced in the presence of fever, hemodynamic instability, uncontrolled pain, severe anemia, or surgical complications (15).

### Outcomes

The primary outcome of this study was pre-chemotherapy postoperative weight loss rate (%), calculated as [(admission weight - pre-chemotherapy weight)/admission weight] × 100. Pre-chemotherapy weight was defined as the last standardized postoperative body-weight measurement obtained before initiation of adjuvant chemotherapy. When chemotherapy was not initiated during the same admission, the last measurement before discharge or before planned chemotherapy assessment was used. Because this clinically relevant time point was not identical in postoperative days for all patients, the outcome should be interpreted as pre-chemotherapy weight loss rather than fixed-day postoperative weight loss.

Secondary outcomes included changes in serum albumin levels, BMI, NRS-2002 score, body composition parameters (including fat-free mass, body fat, skeletal muscle mass, and edema index), handgrip strength, time to initiation of adjuvant chemotherapy, and postoperative length of hospital stay. Time to chemotherapy (TTC) was defined as days from surgery to the first administration of adjuvant chemotherapy.

### Statistical analysis

Continuous variables were expressed as mean ± standard deviation and compared using independent-samples t-tests when approximately normally distributed. Variables not normally distributed were compared using the Mann-Whitney U test and reported with the corresponding Z statistic. Categorical variables were presented as frequencies and percentages and analyzed using chi-square tests or Fisher’s exact tests when appropriate. Ordinal variables, including surgical grading, were compared using the Mann–Whitney U test because the categories reflected increasing levels of surgical complexity from low to high. All statistical tests were two-tailed, and a P value <0.05 was considered statistically significant. Owing to the historical-control design and limited available covariates, results were interpreted as associations rather than causal effects.

## Results

### Participants and baseline characteristics

A total of 160 patients with ovarian cancer undergoing ultra-radical CRS were included in this study, with 80 patients in each group. As shown in **Table 1**, age, BMI, and ordinal surgical grading did not differ significantly between the two groups (all P > 0.05), suggesting comparability in the available baseline variables. However, unmeasured clinical or surgical differences between the historical cohorts could not be fully excluded.

**Table 1:** Demographic and clinical characteristics of patients (n=160).

Characteristics	Control group (n=80)	Experimental group (n=80)	Statistical value (t/χ2/Z)	P-value
Age	58.94±10.92	58.86±10.89	t= 0.043	0.966
BMI	22.89±3.45	22.12±3.17	t= 1.467	0.144
Surgical grading			Z= -0.773	0.440
Low	13	6		
Medium	18	35		
High	49	39		

Surgical grading was treated as an ordinal variable reflecting increasing surgical complexity from low to high and was compared using the Mann–Whitney U test. Continuous variables are presented as mean ± standard deviation.

### Postoperative weight and body composition changes

Postoperative changes in body weight and nutritional status are presented in **Table 2**. The experimental group had a significantly lower pre-chemotherapy postoperative weight loss rate compared to the control group (2.90% vs. 5.31%, P<0.001). In addition, the proportion of patients experiencing clinically significant weight loss (>5%) was significantly lower in the experimental group (31.3% vs. 48.8%, P = 0.024).

**Table 2:** Comparison of postoperative changes in body weight, body composition, and nutritional status between the control and experimental groups (N=160).

Variables	Control group (n=80)	Experimental group (n=80)	Statistics	P-value
Weight loss rate (%)	5.31 ± 4.40	2.90 ± 4.55	t=3.404	<0.001
Weight loss > 5%	39 (48.8%)	25 (31.3%)	χ²=5.100	0.024
BMI change (kg/m²)	-1.27 ± 1.02	-0.73 ± 1.00	t=-3.479	<0.001
Nutritional indicators				
Albumin change (g/L)	-6.98 ± 5.70	-4.10 ± 4.54	t=-3.473	<0.001
NRS-2002 score change	-0.12 ± 2.15	-0.38 ± 2.08	t=0.812	0.418
Body composition change				
Fat-free mass change (kg)	-1.82 ± 3.65	1.96 ± 3.88	t=-3.421	<0.001
Body fat change (kg)	-1.12 ± 4.85	-1.56 ± 5.02	t=0.523	0.601
Skeletal muscle change (kg)	-1.90 ± 4.54	-0.76 ± 2.67	t=-1.927	0.055
Functional & other indicators				
Handgrip strength change (kg)	-0.04 ± 4.20	-0.54 ± 0.67	t=1.148	0.251
Edema index change	-0.02 ± 0.05	-0.04 ± 0.06	t=0.742	0.459

Weight loss rate (%) refers to the pre-chemotherapy postoperative weight loss rate, calculated as [(admission weight − pre-chemotherapy weight) / admission weight] × 100. Approximately normally distributed continuous data are presented as mean ± standard deviation and compared using independent samples t-test; categorical data are presented as number (percentage) and compared using χ² test. All P values are two-tailed.

The decline in BMI was significantly smaller in the experimental group (−0.73 vs. −1.27, P<0.001). Similarly, the reduction in serum albumin levels was significantly attenuated in the experimental group compared to the control group (−4.10 vs. −6.98 g/L, P<0.001). No statistically significant difference was observed in NRS-2002 score changes between the two groups (P = 0.418).

Regarding body composition, fat-free mass showed an apparent increase in the experimental group (1.96 ± 3.88 kg) and a decrease in the control group (-1.82 ± 3.65 kg; P<0.001). Skeletal muscle loss was numerically smaller in the experimental group (-0.76 vs. -1.90 kg), but the corrected two-tailed P value was 0.055. No significant differences were found in body fat change (P = 0.601), handgrip strength change (P = 0.251), or edema index change (P = 0.459).

### Treatment-related time indicators

Treatment-related outcomes are summarized in **Table 3**. The mean TTC was shorter in the experimental group than in the control group (14.84 ± 12.76 vs. 22.03 ± 16.53 days, P = 0.002). Postoperative length of stay also differed statistically (P = 0.022), although the absolute mean difference was modest and should be interpreted in relation to the skewed distribution and discharge practices.

**Table 3:** Comparison of treatment-related time indicators between the two groups (N=160).

Variables	Control group (n=80)	Experimental group (n=80)	Statistics	P-value
Time to first chemotherapy (days)	22.03 ± 16.53	14.84 ± 12.76	t=3.085	0.002
Postoperative length of stay (days)	12.00 (9.00, 14.00)	10.00 (8.00, 13.00)	Z=2.282	0.022

Normally distributed continuous data are presented as mean ± standard deviation and compared using independent samples t-test; non-normally distributed continuous data are presented as median (interquartile range) and compared using Mann-Whitney U test. All P values are two-tailed.

### Implementation and feasibility of the program

Following implementation of the multidisciplinary intervention, the consultation rate for rehabilitation therapists increased from 0% to 85%. In addition, the rate of dietitian involvement increased by approximately 20% in 2025 compared with 2024. These findings indicate good adherence and feasibility of the multidisciplinary intervention model in clinical practice.

## Discussion

This study evaluated a multidisciplinary perioperative intervention targeting postoperative weight loss, nutritional deterioration, and recovery efficiency in patients undergoing ultra-radical CRS for ovarian cancer. The intervention was associated with lower pre-chemotherapy postoperative weight loss, a lower proportion of patients with >5% weight loss, attenuated albumin decline, and shorter TTC. The findings support the feasibility of embedding nutrition and rehabilitation into perioperative nursing-led supportive care, while also highlighting important methodological cautions related to historical controls, limited surgical-complexity characterization, and body composition measurement.

### Mitigation of the postoperative catabolic response

Ultra-radical CRS for advanced ovarian cancer typically involves extensive multivisceral resections that can trigger systemic inflammation, endocrine stress responses, and accelerated skeletal-muscle protein breakdown (8, 20). These mechanisms contribute to UWL, fat-free mass depletion, muscle weakness, and delayed functional recovery. In this study, patients in the intervention group experienced less pre-chemotherapy weight loss and were less likely to lose >5% of body weight. These results are clinically relevant because postoperative weight loss may reduce tolerance to adjuvant chemotherapy and compound the pre-existing nutritional vulnerability of patients with advanced OC (12, 14).

The observed benefit is biologically plausible. Early nutritional assessment can identify inadequate energy/protein intake before visible deterioration occurs, while oral nutritional supplementation, symptom control, and escalation to enteral or parenteral support can reduce the duration of negative nitrogen balance (10, 11). Concurrent rehabilitation may also counteract inflammation-related anabolic resistance by stimulating muscle protein turnover, preserving mobility, and preventing prolonged bed rest (15, 20). Therefore, the program likely acted through multiple mechanisms rather than through weight monitoring alone.

### Preservation of nutritional and metabolic homeostasis

Serum albumin is widely used in surgical oncology as a clinically relevant marker of systemic inflammation, metabolic stress, and overall nutritional risk, although it is not a standalone indicator of nutritional status (20, 21). Following major surgery, albumin levels typically decline due to inflammatory responses, capillary leakage, and reduced hepatic synthesis (22, 23). In this study, the experimental group exhibited a significantly smaller reduction in serum albumin levels, suggesting improved metabolic stability and recovery capacity.

However, no significant difference was observed in NRS-2002 score changes between the two groups. The absence of a between-group difference in NRS-2002 change suggests that screening tools may be less sensitive to short-term postoperative changes than biochemical and body composition indicators. Screening scores are useful for identifying risk, but they may not capture rapid catabolic changes during the early postoperative period (18). Similar discrepancies between objective and screening-based indicators have been reported in previous studies (14). Unlike conventional care, which often relies on reactive nutritional supplementation, our model was supported by a high rate of adherence to nutritionist consultations, enabling earlier identification and proactive management of postoperative nutritional barriers such as malabsorption and ileus. This is in line with previous reports emphasizing that individualized, multidisciplinary perioperative nutrition care, including dietitian-led assessment and early enteral/oral strategies, is safe and may facilitate gastrointestinal recovery and overall postoperative rehabilitation (10, 15).

### Interpreting the fat-free mass finding

The apparent increase in fat-free mass in the experimental group is physiologically unexpected after ultra-radical surgery and should not be interpreted as true postoperative muscle accretion. Bioelectrical impedance analysis estimates fat-free mass partly from body water compartments; therefore, perioperative fluid therapy, occult edema, ascites changes, and redistribution between extracellular and intracellular water may artifactually increase lean-mass estimates (24). Although the edema index did not differ significantly between groups, this does not completely exclude measurement artifact. For this reason, the study conclusion emphasizes weight loss and albumin preservation rather than claiming definitive improvement in lean-tissue accretion. Future studies should use standardized hydration assessment and, ideally, computed tomography-based skeletal muscle area or dual-energy X-ray absorptiometry to validate body composition changes (25).

### Clinical efficiency and chemotherapy timing

The shorter TTC in the experimental group suggests earlier postoperative readiness for adjuvant therapy, but it should not be interpreted as unambiguously superior in oncological terms. Delayed TTC has been associated with worse outcomes in several ovarian cancer cohorts, especially after optimal cytoreduction (26, 27). However, recent evidence indicates that excessively early initiation may not always be beneficial, and an optimal window of approximately 22–35 days has been proposed (28). In the present study, the control group mean TTC of approximately 22 days fell at the lower boundary of that proposed window, whereas the experimental group mean TTC of approximately 15 days was earlier. Therefore, the finding is best interpreted as an indicator of improved short-term recovery capacity and clinical readiness rather than direct evidence of improved survival.

A nuanced interpretation is particularly important in a quality improvement design. The decision to initiate chemotherapy depends on wound healing, bowel function, infection risk, performance status, laboratory recovery, and patient preference. The multidisciplinary pathway may have improved these readiness indicators through earlier nutrition and rehabilitation involvement. Nevertheless, whether earlier TTC translates into better recurrence or survival outcomes cannot be determined from the current dataset.

### Strengths and limitations

This study has several strengths. It addresses a clinically meaningful gap in perioperative supportive care for a high-risk gynecologic oncology population, uses a pragmatic MDT pathway that can be implemented in routine practice, and includes body composition and treatment-related recovery indicators rather than body weight alone.

Several limitations should be considered. First, the quasi-experimental design with non-concurrent historical controls introduces temporal bias. Although institutional surgical, anesthesia, and postoperative routines were intended to remain stable, unmeasured changes in clinical practice, staff experience, referral patterns, or case mix may have influenced the results. Second, the lack of randomization and the limited granularity of surgical-complexity data increase the possibility of residual confounding, even though ordinal surgical grading did not differ significantly between groups. Third, the BMI restriction limits generalizability to underweight, overweight, or obese patients undergoing ultra-radical CRS. Fourth, several clinically important baseline and oncological variables, including FIGO stage distribution, histologic subtype, ascites volume, operative time, blood loss, bowel resection details, neoadjuvant chemotherapy exposure, R0 resection rate, recurrence, and progression-free survival, were not available in the provided quality improvement dataset. Fifth, the primary outcome was assessed at a clinically relevant pre-chemotherapy time point rather than at a fixed postoperative day; therefore, differences in TTC may have influenced the observed magnitude of weight loss. Finally, bioelectrical impedance-derived fat-free mass may be affected by perioperative fluid shifts. Prospective multicenter studies with concurrent controls, standardized surgical complexity scoring, fixed body composition measurement time points, and oncological outcomes are warranted.

## Conclusion

A multidisciplinary perioperative weight management program was associated with lower pre-chemotherapy postoperative weight loss, attenuated albumin decline, and earlier clinical readiness for adjuvant chemotherapy among patients undergoing ultra-radical CRS for ovarian cancer. The findings support the feasibility of integrating nutrition, rehabilitation, and nursing-led monitoring into perioperative gynecologic oncology care. However, causal interpretation is limited by the historical-control design, limited surgical-complexity characterization, restricted BMI range, and lack of long-term oncological outcomes.

## Data Availability

The raw data supporting the conclusions of this article will be made available by the authors, without undue reservation.

## References

[1] Ferlay JE Lam F Laversanne M Colombet M Mery L Piñeros M . Global Cancer Observatory: Cancer Today. Ovary Fact Sheet. Lyon, France: International Agency for Research on Cancer (2024).

[2] Ferlay JE Lam F Laversanne M Colombet M Mery L Piñeros M . Global Cancer Observatory: Cancer Today. China Fact Sheet. Lyon, France: International Agency for Research on Cancer (2024).

[3] Ghose A McCann L Makker S Mukherjee U Gullapalli SVN Erekkath J . Diagnostic biomarkers in ovarian cancer: advances beyond CA125 and HE4. Ther Adv Med Oncol. (2024) 16:17588359241233225. doi: 10.1177/17588359241233225 38435431 PMC10908239

[4] Bryant A Hiu S Kunonga PT Gajjar K Craig D Vale L . Impact of residual disease as a prognostic factor for survival in women with advanced epithelial ovarian cancer after primary surgery. Cochrane Database Syst Rev. (2022) 9:Cd015048. doi: 10.1002/14651858.cd015048 36161421 PMC9512080

[5] Lee EJ Park SJ Kim HS . Splenectomy and distal pancreatectomy in advanced ovarian cancer. Gland Surg. (2021) 10:1218–29. doi: 10.21037/gs-2019-ursoc-09 33842268 PMC8033062

[6] Norppa N Staff S Helminen M Auranen A Saarelainen S . Improved survival after implementation of ultra-radical surgery in advanced epithelial ovarian cancer: Results from a tertiary referral center. Gynecologic Oncol. (2022). doi: 10.1016/j.ygyno.2022.03.023 35397919

[7] Aletti GD Dowdy SC Podratz KC Cliby WA . Relationship among surgical complexity, short-term morbidity, and overall survival in primary surgery for advanced ovarian cancer. Am J Obstet Gynecol. (2007) 197:676.e1–7. doi: 10.1016/j.ajog.2007.10.495 18060979

[8] Cole CL Kleckner IR Jatoi A Schwarz EM Dunne RF . The role of systemic inflammation in cancer-associated muscle wasting and rationale for exercise as a therapeutic intervention. JCSM Clin Rep. (2018) 3(2):e00065. doi: 10.17987/jcsm-cr.v3i2.65 31134216 PMC6534125

[9] Obermair A Simunovic M Isenring L Janda M . Nutrition interventions in patients with gynecological cancers requiring surgery. Gynecologic Oncol. (2017) 145:192–9. doi: 10.1016/j.ygyno.2017.01.028 28173966

[10] Weimann A Braga M Carli F Higashiguchi T Hübner M Klek S . ESPEN practical guideline: Clinical nutrition in surgery. Clin Nutr. (2021) 40:4745–61. doi: 10.1016/j.clnu.2021.03.031 34242915

[11] Muscaritoli M Arends J Bachmann P Baracos V Barthelemy N Bertz H . ESPEN practical guideline: Clinical nutrition in cancer. Clin Nutr. (2021) 40:2898–913. doi: 10.25284/2519-2078.2(103).2023.284622 33946039

[12] Wu Y Mu J Cao J Li D Dai Y . Research status and progress of nutritional support therapy for ovarian cancer. Nutr Cancer. (2021) 74:1519–26. doi: 10.1080/01635581.2021.1957132 34323140

[13] Nasser S Bilir E Derin X Richter R Grabowski JP Ali P . Pre-operative malnutrition in patients with ovarian cancer: what are the clinical implications? Results of a prospective study. Cancers. (2024) 16:622. doi: 10.3390/cancers16030622 38339372 PMC10854561

[14] Shao Z Fu L Lu Y Zhou H Zhu Y Zhu T . Ovarian cancer and malnutrition: a literature review. Trans Cancer Res. (2025) 14:3239–54. doi: 10.21037/tcr-2025-758 40530143 PMC12169986

[15] Gillis C Hasil L Kasvis P Bibby N Davies SJ Prado CM . Nutrition care process model approach to surgical prehabilitation in oncology. Front Nutr. (2021) 8. doi: 10.3389/fnut.2021.644706 34249985 PMC8264148

[16] Taylor MJ McNicholas C Nicolay C Darzi A Bell D Reed JE . Systematic review of the application of the plan-do-study-act method to improve quality in healthcare. BMJ Qual Saf. (2014) 23:290–8. doi: 10.1136/bmjqs-2013-001862 24025320 PMC3963536

[17] Hoffmann TC Glasziou PP Boutron I Milne R Perera R Moher D . Better reporting of interventions: template for intervention description and replication (TIDieR) checklist and guide. BMJ. (2014) 348:g1687. doi: 10.1136/bmj.g1687 24609605

[18] Kondrup J Rasmussen HH Hamberg O Stanga Z Ad Hoc Working Group ESPEN . Nutritional risk screening (NRS 2002): a new method based on an analysis of controlled clinical trials. Clin Nutr. (2003) 22:321–36. doi: 10.1016/s0261-5614(02)00214-5 12765673

[19] Cederholm T Jensen GL Correia MITD Gonzalez MC Fukushima R Higashiguchi T . GLIM criteria for the diagnosis of malnutrition - a consensus report from the global clinical nutrition community. Clin Nutr. (2019) 38:1–9. doi: 10.1016/j.clnu.2018.08.002 30181091

[20] Ljungqvist O Weimann A Sandini M Baldini G Gianotti L . Contemporary perioperative nutritional care. Annu Rev Nutr. (2024) 44:231–55. 10.1146/annurev-nutr-062222-021228 39207877

[21] McClave SA Martindale RG Vanek VW McCarthy M Roberts P Taylor B . Guidelines for the provision and assessment of nutrition support therapy in the adult critically ill patient: Society of Critical Care Medicine (SCCM) and American Society for Parenteral and Enteral Nutrition (A.S.P.E.N.). JPEN J Parenteral Enteral Nutr. (2016) 33:277–316. doi: 10.1177/0148607109335234 19398613

[22] Soeters PB Wolfe RR Shenkin A . Hypoalbuminemia: pathogenesis and clinical significance. JPEN J Parenter Enteral Nutr. (2019) 43:181–93. doi: 10.1002/jpen.1451 30288759 PMC7379941

[23] Allison SP Lobo DN . The clinical significance of hypoalbuminaemia. Clin Nutr. (2024) 43:909–14. doi: 10.1016/j.clnu.2024.02.018 38394971

[24] Ræder H Kværner AS Henriksen C Florholmen G Henriksen HB Bøhn SK . Validity of bioelectrical impedance analysis in estimation of fat-free mass in colorectal cancer patients. Clin Nutr. (2018) 37:292–300. 28122662 10.1016/j.clnu.2016.12.028

[25] Ugras S . Evaluating of altered hydration status on effectiveness of body composition analysis using bioelectric impedance analysis. Libyan J Med. (2020) 15:1741904. doi: 10.1080/19932820.2020.1741904 32182203 PMC7144212

[26] Sp S C RK Kr A Y R N AK Fernandez AM . Delay in time to adjuvant chemotherapy and its impact on oncological outcomes in patients undergoing optimal cytoreductive surgery for advanced ovarian cancer: analysis of 1480 cases from the Indian HIPEC Registry. J Surg Oncol. (2024) 130(6):1358–63. doi: 10.1002/jso.27896 39348465

[27] Chetverikov S Zavoloka S Chetverikov M Chetverikova-Ovchynnyk V . The interval between the primary cytoreductive surgery and adjuvant chemotherapy in patients with advanced ovarian cancer. Med Res J. (2020). doi: 10.5603/mrj.a2020.0027

[28] Meyer-Wilmes P Kennes LN Ignatov A Goetz F Wittenborn J Stickeler E . Earlier is not always better: optimal time to initiate adjuvant chemotherapy after surgery for ovarian cancer. Arch Gynecology Obstetrics. (2025) 312:1163–73. doi: 10.1007/s00404-025-08095-3 40643609 PMC12414076

